# Supplementary material for: Magnetoelectrical control of nonreciprocal microwave response in a multiferroic helimagnet
Source: Nat Commun. 2017 May 8;8:15252. doi: 10.1038/ncomms15252 (PMC5424162; doi:10.1038/ncomms15252)
Supplement: Supplementary Information — Supplementary Figures [file ncomms15252-s1.pdf]

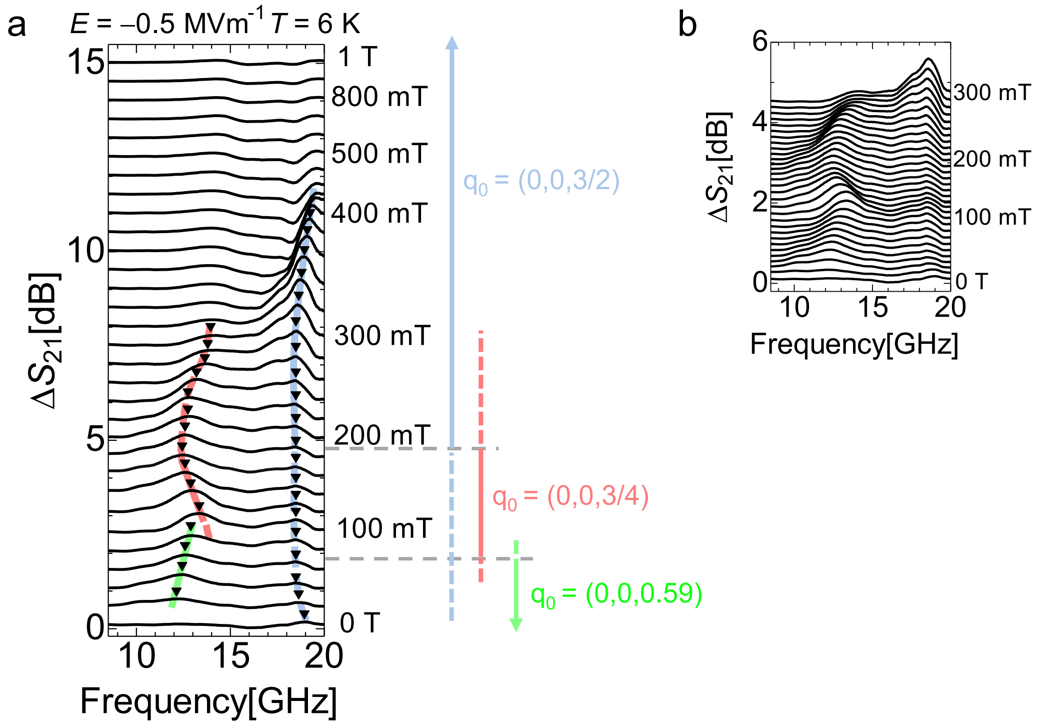

Supplementary Figure 1: Details of magnetic field dependence of microwave absorption spectra. (**a**) Data from 0 to 1 T measured at 6 K. (**b**) More detailed data in the low magnetic field region.

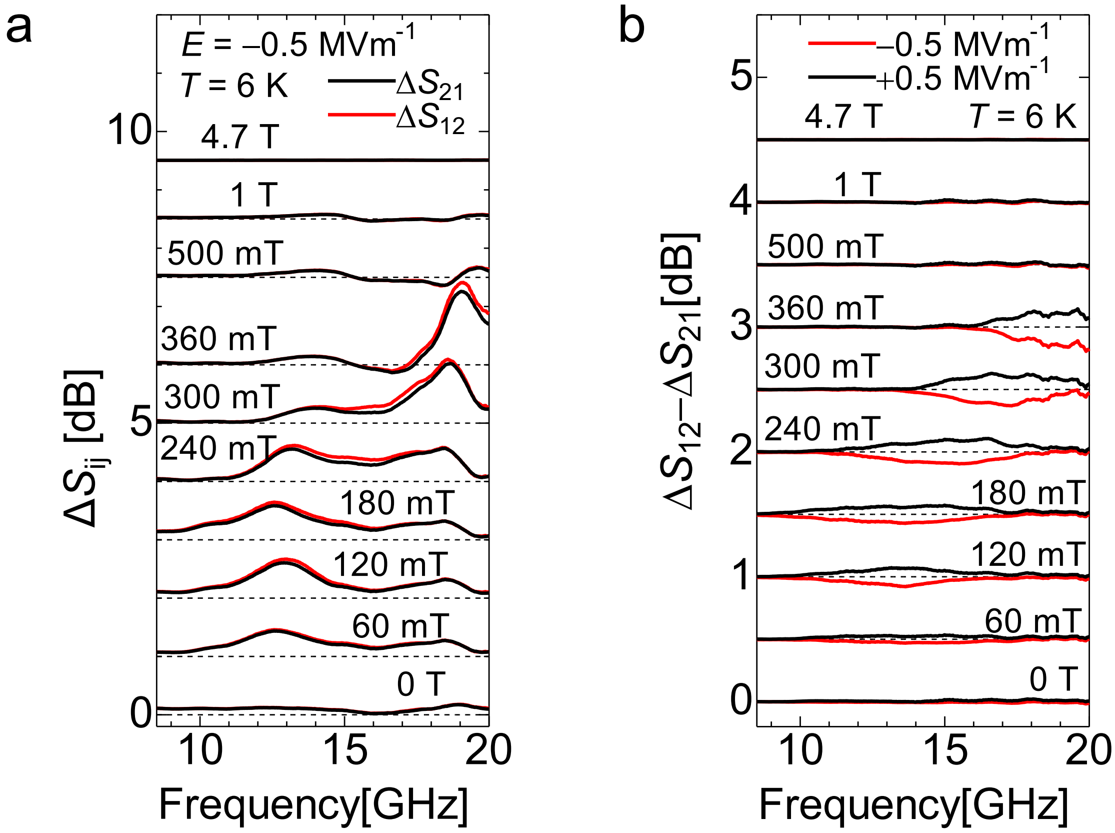

Supplementary Figure 2: **Details of magnetic field dependences of microwave nonreciprocity.** (**a**) magnetic field dependence of microwave absorptions ΔS_{12} and ΔS_{21} at 6 K measured after the poling with $E = -0.5$ MVm^{-1}. (**b**) Magnetic field dependences of microwave nonreciprocity $\Delta S_{12} - \Delta S_{21}$ at 6 K measured after the poling with $E = \pm 0.5$ MVm^{-1}, respectively.
